# Effect of enamel deproteinization with 5.25% sodium hypochlorite on the bond strength of orthodontic brackets. An experimental study

**DOI:** 10.4317/jced.61807

**Published:** 2024-08-01

**Authors:** Ana-Carolina Mas-López, Julissa Robles-Ruíz, Luis-Ernesto Arriola-Guillén

**Affiliations:** 1 Orthodontic student, School of Dentistry, Universidad Científica del Sur, Lima, Perú; 2 MSc and Associate Professor of the Division of Orthodontics, Universidad Científica del Sur, Lima, Perú; 3 Ph.D. and Associate Professor of the Division of Orthodontics, Universidad Científica del Sur, Lima, Perú

## Abstract

**Background:**

In the search for alternatives to increase the bond strength of brackets, when necessary, this study proposes to apply the enamel deproteinization protocol to eliminate the proteins in the surface enamel to achieve better etching patterns and thereby increase bond strength. The aim of this study was to evaluate the effect of sodium hypochlorite as a deproteinizing agent on the bond strength of metal brackets.

**Material and Methods:**

Forty bovine teeth were randomly and equally divided into two groups. The experimental group (n=20) underwent a deproteinization treatment with sodium hypochlorite at 5.25% for 60 seconds prior to acid etching of the enamel with 37% phosphoric acid and bracket bonding with Transbond XTTM resin. The control group (n=20) underwent enamel etching with 37% phosphoric acid and bracket bonding with Transbond XTTM resin. Shear strength and the adhesive remnant index were evaluated and the Students t and Chi-square tests were used (*P*<0.05).

**Results:**

The bond strength values of the control group (27.72±6.42 Mpa) were not significantly lower compared to the experimental group (29.21± 7.96 Mpa) (*p*=0.259). The adhesive remnant index showed a similar behavior in both groups, with the amount of adhesive remaining on the enamel being less than 50% in most samples of both the control and experimental group.

**Conclusions:**

Deproteinization treatment of bovine tooth enamel with 5.25% sodium hypochlorite for 60 seconds prior to enamel acid etching does not improve the bond strength of a resin in orthodontic bracket bonding.

** Key words:**Deproteinization, sodium hypochlorite, bracket bonding, orthodontics.

## Introduction

The bonding of brackets to tooth enamel is an important step in orthodontic treatment because they must remain in position for a long time and be exposed to chewing, orthodontic and other forces. Nowadays, in most cases, the bonding materials for brackets provide adequate bonding forces (1). However, in some circumstances it is necessary to reinforce the bonding, as in the case of patients with limited oral opening, bonding in teeth with structural alterations of the enamel, such as hypocalcification or fluorosis; clinical conditions which, due to the difficulty of access, require a technique that allows greater adhesion, such as the sticking of tubes in second molars with poor position and difficult access, retained or impacted teeth that need to be tractioned or also in some cases of indirect bonding and lingual orthodontics; clinical situations in which the detachment of brackets continues to be a problem. This challenge of reinforcing bonding has given rise to multiple alternatives to increase adhesion, including sandblasting of the enamel prior to acid etching, pretreatment of the enamel with low frequency laser, and a procedure little studied in the field of orthodontics, which is the deproteinization of the enamel (2-4).

It has been found that the effect of acids on tooth enamel can change. Silverstone *et al*. (5) demonstrated that the action of acidic solutions does not produce a specific etching pattern on the enamel surface, on finding that acids produced 3 types of etching patterns. Of these etching patterns, types 1 and 2 offer the greatest area and depth of retention while the type 3 pattern is the least favorable. These three patterns appear randomly at any point on the enamel, but clinically only an opaque white surface is observed showing the quantity but not the quality of the etched surface (6). These acid-produced differences could be due to variation in chemical composition and crystal orientation, highlighting the variations present in enamel structure and that they can occur not only from tooth to tooth, or surface to surface, but also from site to site on a single tooth surface. Kelly *et al*. (7) reported differences in enamel surface structure observed by scanning electron microscopy, associated with genetic variants; concluding that this variation can affect the formation of enamel structure.

Espinosa *et al*. (8) evaluated the effect of enamel deproteinization on acid etching. They topographically evaluated the surface of deproteinized enamel etched with phosphoric acid, compared with enamel treated only with acid etching, and concluded that the pretreatment of enamel with sodium hypochlorite at 5. 25% for 60 seconds prior to acid etching increases the type 1 and 2 etch patterns, providing better retention areas in size and depth by removing organic matter from the enamel surface of both the acquired film and the enamel structure. Several chemical reactions occur when sodium hypochlorite (NaClO) is in contact with organic matter, resulting in liquefaction of the organic tissues and generating better conditions for bonding (9).

Therefore, in the search for alternatives to increase bond strength, this study proposes the application of the enamel deproteinization protocol suggested by Espinosa *et al*. (8) for the elimination of proteins in the surface enamel, with the aim of achieving better etching patterns and, thus, increasing the bond strength of a resin in orthodontic bracket bonding. The present study aimed to evaluate this procedure which has been little studied and used in the field of orthodontics. Consequently, the purpose of this study was to compare the bond strength of metal brackets bonded to bovine enamel treated with and without deproteinization of the enamel with 5.25% NaClO prior to acid etching.

## Material and Methods

This experimental *in vitro* study on bovine teeth was approved by the technical report of the reviewers of the Stomatology Career of the Universidad Científica del Sur with code No. 321-POS-2017.

The sample consisted of 40 recently extracted bovine incisors, free of caries, fractures, wear, or any other visible defect in the enamel. The incisors were extracted from the jaws of cattle slaughtered for human consumption, were washed with abundant drinking water, and the remains of adhered tissues were eliminated. They were then cut transversely at the neck level separating the crown from the root. To eliminate the roughness of the vestibular surface, a 1 mm abrasion was made using 600 grit sandpaper that was placed in a lathe, obtaining a completely flat, smooth surface free of irregularities, and they were immediately stored in distilled water at room temperature.

**Figure 1:**
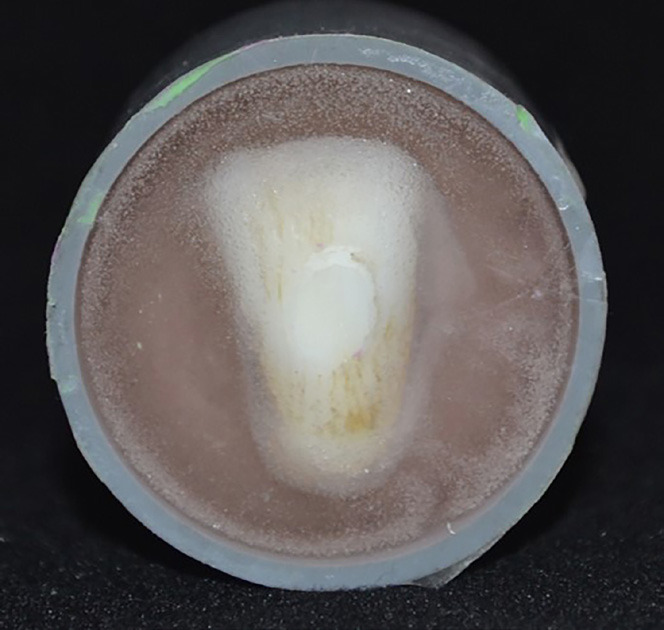
Specimen dipped in acrylic resin, with the enamel surface free for adhesion.

Subsequently, the teeth were immersed in acrylic resin placed in PVC tubes of 1¼” diameter by 40 mm in height, leaving the flat surface of the enamel free (Fig. 1).

-Bracket bonding protocol

Forty lower incisor metal brackets with 0.022” Edgewise Slim slot (Morelli Ortodontia, Sorocaba - SP Brazil) were used. The area of the bracket base was calculated using a microscope, and the value obtained was 9.055 mm2.

The teeth were divided equally and randomly into two groups (20 in each group) in which two different bonding protocols were applied. Prior to each bonding procedure the teeth were cleaned and polished with a prophylaxis brush and pumice paste with water for 10 seconds, rinsed for 10 seconds and dried with air from a triple syringe.

-Group 1 (Control group) 

**Figure 2:**
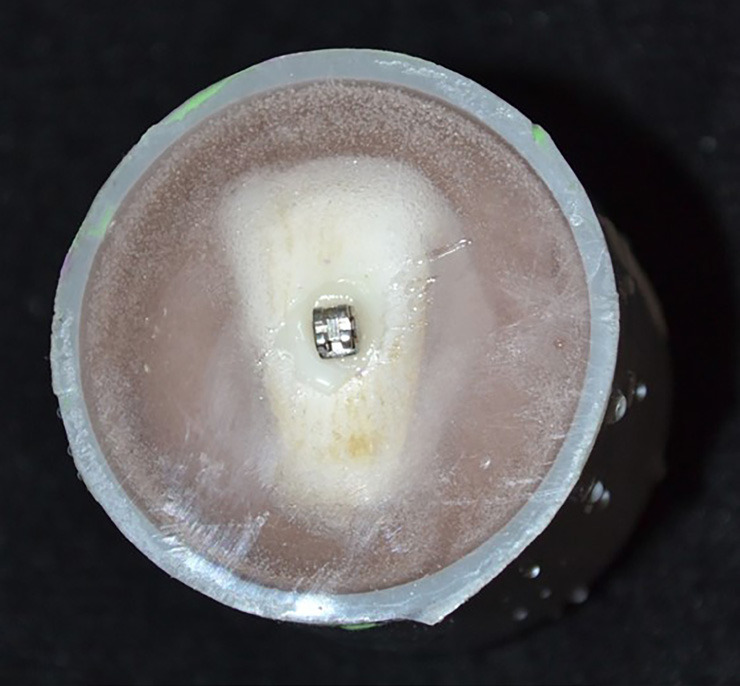
Bracket bonded to enamel surface.

The bonding protocol indicated by the manufacturer was used; 37% phosphoric acid (3M ESPE Scotchbond etchant) was applied and the enamel surface was etched for 30 seconds, washed with abundant pressurized water for 20 seconds and dried with air. Then, a thin layer of Transbond XTTM primer was applied on the etched surface with a brush and a small amount of Transbond XTTM resin was applied on the base of the bracket. The bracket was placed on the surface of the tooth and pressed firmly, applying a force of 380 grams using a dynamometer (Orthosur, Brazil) to standardize the thickness of the resin, the excess around the base of the bracket was removed using a scaler and light cured for 40 seconds (10 seconds on each side of the bracket) using an EliparTM S10 LED curing light (3M ESPE) (Fig. 2). The specimens were immediately stored again in distilled water at room temperature until use.

-Group 2 (Experimental group)

Before applying the bonding protocol indicated by the manufacturer, the enamel deproteinization procedure was performed prior to acid etching, for which 5.25% NaClO was applied with a microbrush performing circular movements for 60 seconds on the surface of the enamel. The enamel was then washed with abundant pressurized water for 20 seconds, and immediately thereafter, the acid etching and cementing of the bracket was performed following the same protocol that was applied in the control group. The specimens were then stored in distilled water until use.

-Shear bond strength test (SBS)

**Figure 3:**
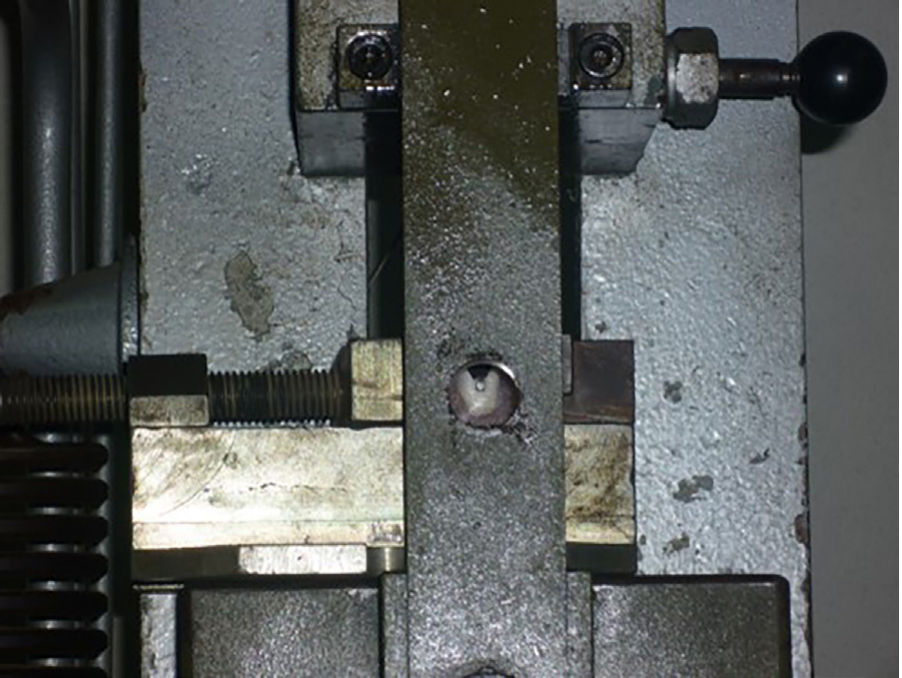
Specimen placed in a press on the universal mechanical testing machine to undergo the shear test.

Bond strength was evaluated in Mpa by measuring shear strength, using a universal mechanical testing machine (Amsler and CIA. Schaffhausen, Switzerland), to which a steel shank with a bevel termination was attached. The specimens were held by a press, oriented in such a way that the bevel applied a load between the base of the bracket and the surface of the tooth parallel to it and in the incisal-gingival direction (Fig. 3). At a speed of 1 mm/min until the bracket detached, this value was recorded in kgF which was then converted to MPa, multiplying the load by 9.8066 and dividing the value obtained by the area of the base of the bracket (9.055mm2).

-Evaluation of the adhesive remnant index (ARI)

The amount of adhesive remnant was quantified by stereo microscopy (10x magnification) observing the amount of adhesive paste remaining on the enamel surface after bracket detachment. Adhesive-free surfaces were recorded as 0, surfaces with up to 50% adhesive remnant were recorded as 1, surfaces with more than 50% adhesive remnant were recorded as 2, and surfaces on which all the adhesive was impregnated were recorded as 3.

Statistical analysis

The data were processed and analyzed using IBM SPSS Statistics version 29 (IBM Corp., NY, USA). For the strength of bonding, the values of central tendency and measures of dispersion were calculated for each group. To determine whether the groups had a normal distribution, the Shapiro Wilk test was applied; since both groups showed normal distribution, the Student’s t-test was applied to determine the difference between the two groups. Likewise, the chi-square test was used to determine if any association was present in the ARI scores in both groups. A significance level of 5% was considered.

## Results

The descriptive statistics of the bond strength values for the control and experimental groups are shown in  Table 1. The mean value found in the experimental group (29.21± 7.96 Mpa) was slightly higher than that of the control group (27.72±6.42 Mpa), with no statistically significant difference between the two groups (*p* = 0.259).

Our study did not find a significant association in the Adhesive Remnant Index (ARI) scores between the groups (*p*=0.916), as indicated in  Table 2. Both groups showed similar behavior, with less than 50% of adhesive remaining on the enamel. The highest percentage of ARI score was 1, with 40% in the control group and 50% in the experimental group.

## Discussion

The purpose of this study was to determine if the deproteinization of enamel with 5.25% sodium hypochlorite applied for 60 seconds before acid etching with 37% phosphoric acid produces an increase in bond strength using the Transbond XTTM system marketed worldwide for orthodontic bonding.

This *in vitro* study was performed in bovine teeth based on multiple studies that used bovine enamel as an alternative to human teeth. The similarity in enamel microstructure of both species, makes bovine teeth an excellent alternative to human teeth in dental research (10-15).

The shear strength values obtained in our study were 29.21± 7.96 Mpa in the NaClO + Transbond XTTM treated group (experimental group) and 27.72±6.42 Mpa in the Transbond XTTM group (control group), with the results of both groups being above the accepTable clinical values (5.9 - 7.8 MPa) proposed by Reynolds *et al*. (1). The bond strength values obtained coincided with the values of 29.19±6.1 MPa reported by Zheng *et al*. (16) using conventional acid etching and Transbond XTTM.

Regarding the effect of 5.25% NaClO prior to enamel acid etching, the present study found no significant increase in the shear strength of the brackets bonded with Transbond XTTM. This is in agreement with the findings of Justus *et al*. (2) and Mahmoud *et al*. (3) who, in the groups in which they used Transbond XTTM resin, found no significant differences in bond strength between the specimens that were treated with NaClO prior to acid etching and those that were not.

Likewise, our results agree with those obtained in the split-mouth trial by Peloso *et al*. (17), who found that enamel deproteinization had no impact on the number of bracket detachments. However, some studies have found different results to those mentioned above. In this sense, Panchal *et al*. (9) reported that enamel treatment with deproteinizing agents including 5.25% NaClO and 10% papain gel significantly increased shear strength This difference in results could be attributed to the shorter acid etching time applied, which was 15 seconds. Also, Sharma *et al*. (18) concluded that deproteinization using 5.25% NaOCl before acid etching significantly increased the shear strength of brackets bonded to teeth with fluorosis. This difference with most of the results reported could be related to the different substrate used, since enamel with fluorosis has a more acid-resistant surface and a significantly higher protein content than healthy enamel.

Contrary to the results reported in previous scientific literature, the study by Huilcapi *et al*. (4) found that the application of NaClO produced a significant decrease in the bond strength of brackets on healthy enamel, attributing this result to the possible presence of free radicals formed by NaClO that can inhibit adequate polymerization of the resin cement, manifesting as a decrease in bond strength. It is essential to consider that the unusual result could be because they did not wash with water after using NaClO before the acid etching. There might have been remaining NaClO, which inhibited proper polymerization.

On the other hand, the ARI scores show that, in most of the specimens of both groups, adhesive failure occurred at the adhesive - enamel interface, which is favorable since, with less residual adhesive remaining on the surface of the tooth, the possibility of damaging this structure at the time of removing the resin remnants after bracket removal decreases.

Finally, after analyzing previous publications on the subject and our results, we do not suggest including the enamel deproteinization protocol prior to acid etching in the bonding of brackets with Transbond XTTM resin, since this would add a step to the bonding procedure without greater clinical benefit, in addition to taking additional precautions for the clinical management of HClO.

## Conclusions

Deproteinization treatment of bovine tooth enamel with 5.25% sodium hypochlorite for 60 seconds prior to enamel acid etching does not significantly improve the bond strength of a resin in bracket bonding.

## Figures and Tables

**Table 1:** Comparison of bond strength in Mpa of brackets bonded to bovine enamel with and without deproteinization treatment with 5.25% NaOCl prior to enamel acid etching.

Group	n	Mean	SD	P-value	Mean difference	95% confidence interval	
						Lower limit	Upper limit
Control group	20	27.72	6.42	0.259	-1.49	-6.12	3.14
Experimental group	20	29.21	7.96				

Student t-test for independent samples SD: standard deviation

**Table 2:** Adhesive remnant index (ARI) in the groups evaluated.

Groups		Index ARI				
		ARI 0	ARI 1	ARI 2	ARI 3	Total
Control group	n	6	8	3	3	20
	%	30.00	40.00	15.00	15.00	100.00
Experimental group	n	5	10	2	3	20
	%	25.00	50.00	10.00	15.00	100.00
Total	n	11	18	5	6	40
	%	27.50	45.00	12.50	15.00	100.00

*P*= 0.916, Chi-square test.

## Data Availability

The datasets used and/or analyzed during the current study are available from the corresponding author.
